# Cognitive CAPTCHA Password Reminder

**DOI:** 10.3390/s23063170

**Published:** 2023-03-16

**Authors:** Natalia Krzyworzeka, Lidia Ogiela, Marek R. Ogiela

**Affiliations:** 1 Cryptography and Cognitive Informatics Laboratory, AGH University of Science and Technology, 30 Mickiewicza Ave, 30-059 Krakow, Polandmogiela@agh.edu.pl (M.R.O.); 2 Institute of Computer Science, AGH University of Science and Technology, 30-059 Krakow, Poland

**Keywords:** personal CAPTCHA, visual CAPTCHA, login scheme, password reminder, cognitive processes, password management

## Abstract

In recent years, the number of personal accounts assigned to one business user has been constantly growing. There could be as many as 191 individual login credentials used by an average employee, according to a 2017 study. The most recurrent problems associated with this situation faced by users are the strength of passwords and ability to recall them. Researchers have proven that “users are aware of what constitutes a secure password but may forgo these security measures in terms of more convenient passwords, largely depending on account type”. Reusing the same password across multiple platforms or creating one with dictionary words has also been proved to be a common practice amongst many. In this paper, a novel password-reminder scheme will be presented. The goal was that the user creates a CAPTCHA-like image with a hidden meaning, that only he or she can decode. The image must be in some way related to that individual’s memory or her/his unique knowledge or experience. With this image, being presented each time during logging in, the user is asked to associate a password consisting of two or more words and a number. If the image is selected properly and strong association with a person’s visual memory has been linked to it, the chances of recalling a lengthy password he/she created should not present a problem.

## 1. Introduction

A study from 2020 shows that four out of five data breaches are caused either by stolen credentials or brute force attacks [1,2,3]. Phishing and man-in-the-middle attacks are the most common ways for hackers to steal logins and associated passwords. Many companies have been fighting against these attacks by making employees or customers aware of scamming methods as well as teaching them how to recognise phishing attempts.

Website providers also have been trying to stop brute force attacks by limiting the number of times users can provide incorrect passwords during login and by linking password recovery processes to a person’s e-mail address. This solution, however, does not work for users who are using popular phrases as passwords (e.g., ‘qwerty’) or common dictionary words. This is because hackers can apply the same ‘popular’ password to many different profiles. If any of these accounts is using this popular phrase, the account’s security is breached.

As has been shown, many users, despite being aware of how unsafe their password may be, will still use it on many different personal accounts for convenience reasons [2,4]. There is really no easy way to combat this practice. The two main reasons behind password reuse are as follows: remembering different passwords for each account or going through a lengthy ‘remind password’ process every time we are asked to login. Password managers appear to be especially effective in such situations, but installing them on each of our electronic devices and paying additional fees for this kind of software does not seem to be an ideal solution for all. Internet users, on average, use the option ‘remind password’ around 22 times per year [5]. This proves that despite their experience and knowledge, most people still overestimate their ability to remember passwords or simply no longer try to remember them, especially for sites they are accessing rarely, and would rather use the option ‘remind password’ each time. Looking from the service provider’s perspective, restoring a user’s forgotten credentials ideally should not generate too many additional costs, yet, for example, sending SMS or e-mail messages to a linked account sometimes can turn out to be costly for a small business. Additional concerns arising from this is what do when an e-mail account or phone has also been stolen or has been changed? It could result that in such cases the account would remain locked forever [6].

When coming up with a solution to solve authentication-related problems, it is important to know the limitations of electronic devices and be aware of the most recent advances in technology.

A couple of years ago, login relying on our facial features or fingerprint would be considered very secure. Currently, with various facial filters (which could help imitate someone else) and the abilities of common devices (which rarely have fingerprint readers), both of these options no longer seem to be safe to use. Authorization based on login credentials and password is very practical and easy to implement; however, there have not been many improvements to this method. Password requirements are getting more complex, and with the constant number of growing accounts, solutions to solve this problem are in demand.

The purpose of this paper is to present a new password generation and reminder protocol that uses CAPTCHA authorization codes and is oriented to a particular system user. These methods will have the following properties:

- The proposed protocol will enable the secure storage of passwords and easier recovery of passwords.
- Passwords will be created using personalized image patterns, as well as appropriate reminder questions.
- The protocol presented will be based on the experience and knowledge of the user in question.
- Password reminders will be based on using the user’s memories, experiences, or cognitive associations.

The paper will be organized in following manner. In Section 2, the password management techniques will be presented. Comparison of our protocol with other solutions will be presented in Section 3. Section 4 will present the CATCHA Password Reminder protocol. Security analysis will be performed in Section 5. Finally, in Section 6 concluding remarks and future research direction will be presented.

## 2. Password Management Techniques

Every authentication process has to be fast, complete, and easy to implement. Yet online authentication cannot rely on ID or cards with microchips, so the identity has to be proven using the user’s own memory and knowledge. The current, most common password recommendations suggest that the password has to be at least eight characters long, with capital and small letters as well as at least one digit [7]. However, the higher the entropy of the password, the more difficult it becomes for the average user to remember it. Therefore, on which solutions are users relying in order not to compromise their account security with trivial passwords?

It is estimated that the reuse of passwords is so common in every age group that around 59–76% of users admit to this kind of practice. Another solution to manage passwords is to write them down on a piece of paper. This, however, requires constant access to that note, which does not make it very practical. Likewise, around 10% of people admit to storing their login credentials in a non-protected text file somewhere on their PC or in their e-mail in drop box (7%). Finally, 28% of users rely completely on password managers [8]. This last option, although very convenient, has some clear downsides. Password managers are required to be installed on every device from which we will be logging in, and they could become a potential target in the case of a hacker attack. Moreover, finding vulnerabilities in newly created password manager apps could be extremely damaging for users, and such bugs are not uncommon. In addition, this kind of service costs from 10USD to 178USD per year, which is an additional cost that users probably would prefer to avoid. Thus, there is truly no optimal universal solution to manage passwords that would suit every user.

If all of the mentioned solutions to manage passwords fail, the user has to have an option to remember the password. As a disadvantage of this step, we can say that it would take the user much more time to complete the authentication procedure. It would generate additional costs for the service provider (e.g., sending SMS, emails) and require an accessible email account or phone number. Moreover, the ‘remind password’ option usually causes a password reset, meaning that the user has to come up on the spot with another set of characters/phrases that he or she most probably will also not remember next time.

In an attempt to help solve problems with multiple password management, a different approach to the login scheme will be introduced in this article. A method described in the next chapter is designed to serve as a password reminder; it is no different than a normal login procedure, except for an additional display of the user’s pre-defined image.

## 3. Similar Works

There have been many attempts to strengthen the user’s memory of his/her login credentials. An attempt shown by the research team in [9] describes a technique of chunking a username and two different types of password (a multiword passphrase and a standard password) and playing a three-round word scramble game with them. Experiments showed that the effect on memory depended on the type of password. “Chunking had a negative impact on memory for multiword passphrases but a positive impact on memory for standard passwords”.

A different attempt, also performed by the authors of this article, was done with the use of Google Street View^®^ image as a password reminder [10]. In this example, the user was asked to set his password as one of the geographical locations. The reminder of that password, shown also during each login, would be a photo of Google Street View^®^ from the said location. The success of this method lies only in the account owner’s ability to pick a very remote/not well-known street and create a password related to it. If the location is picked correctly, there is really only a slim chance of performing a reverse Google Image search due to the size of the Google Maps^®^ database. To further strengthen this method, the authors suggested cropping the street view or taking photos of a part of a building, so as to further reduce any chances of recognition.

A study done by Nielsen Norman Group [11] summarises some of the techniques to help users quickly recall their login credentials. It notes that all information remembered by humans goes through the following stages: encoding, storage, and retrieval. The article points out that the weakest link here is the encoding part, and more focus needs to be put to strengthen it. Researchers note that more attention needs to be paid when creating a password. Users need to concentrate on this part rather than treat it as a hurdle and rush through it. The following advice was given in the article summary: eliminate distractions, pay more attention when creating passwords, choose memorable passwords, do a word play on the password, reset the password when you are not busy/distracted with other work, create your own password guidelines, and, for service providers, add tips to the webpages.

## 4. CAPTCHA Password Reminder Protocol

The goal of the presented scheme is to help the user recall his/her password by showing them an image. In order for this to happen, the said image must carry some kind of a secret meaning to the account owner. The described image–password link has to be established by the user earlier, at the stage of password creation, by associating a scene presented on the graphic with the user’s memory, experience, or specific knowledge. This association, however, cannot be too easy to guess, so as not to compromise the account’s safety.

The CAPTCHA password reminder scheme account creation and authentication process is as follows:

- First, during the password (or account) creation, the user is asked to upload a graphic image or to pick one from a selection given by the service provider.
- After the image has been uploaded, the account owner is asked to create a password that is directly or indirectly associated with that image. It is important to stress to the user that the image could also serve as a clue to potential hackers, so the password should not be possible to decrypt without their own experience or knowledge.
- In order to further increase the password entropy, the next step in creating the CAPTCHA password reminder scheme is to pick a so-called standard password reset question. This question also needs to be chosen by the user during their account creation or password change, and the response to it has to be somehow incorporated into the password itself. This step was chosen simply because responses to typical password reset questions are also very personal and hard to guess by a random person, which makes it a perfect addition to the password.
- To guarantee that the user does not forget about all of the parts of his/her password (the image part, and the reset question part), password reset questions will appear embedded on the image in CAPTCHA-like letters.

The pattern that the user’s brain will follow after seeing the CAPTCHA password reminder scheme image must be complex, appear random to others, and rely on a piece of information that only the account owner would know. An example would be a photo of a neighbour’s dog or a secret location. Providing names of both should not be at all difficult for the account owner, but to a stranger those images should be meaningless. It would also be a good idea to upload an image that contains multiple objects and associate the password only with one random image section (e.g., a movie scene in which our focus would be only on the actor’s shoes). Created correlations could go as far as the user decides (e.g., image of a CD cover from a band we like, linked to a memory of our vacation trip, during which a song from that CD was played, and then picking the name of our destination as a password). Possibilities of these image–password associations are endless and, when well thought through, could guarantee enough safety that guessing a password for an image would be impossible. 

**Figure 1:**
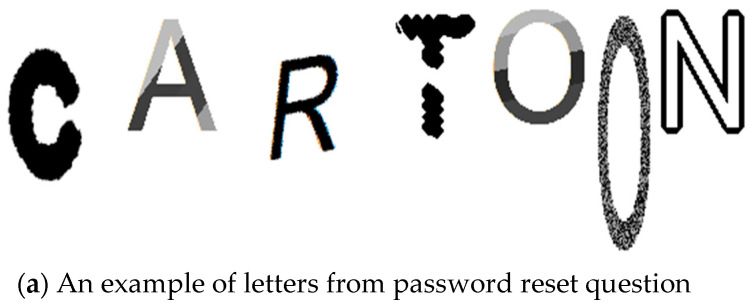

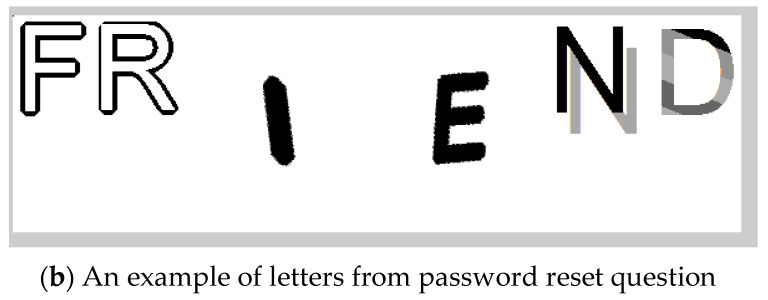
Part of password reset questions, changed to CAPTCHA letters.

**Figure 2:**
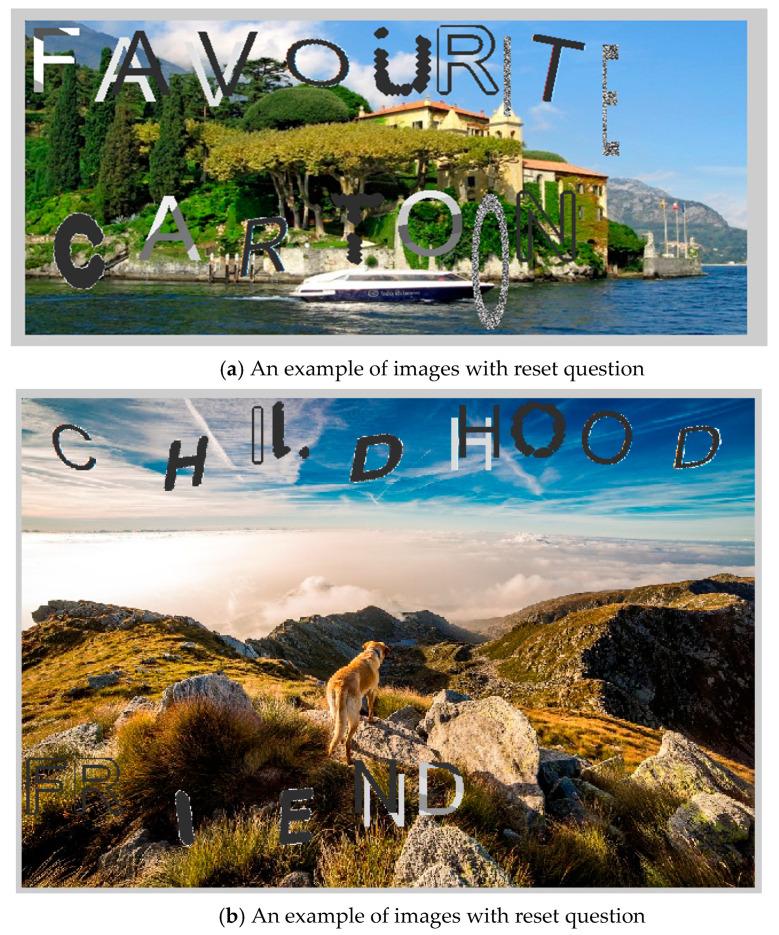
CAPTCHA password reminder images with password reset questions embedded into them.

The algorithm that generates CAPTCHA-like letters was presented in [12]. It relies on various image processing methods, such as dilatation and skeletonization. The CAPTCHA letter generation algorithm was chosen in hopes of preventing hackers from finding similar images on the web and to block boots from reading reset questions. After processing, each letter or reset question has a different style and size. Before and after the result of embedding the reset, a question is presented in Figure 1 and Figure 2. Thus, the generated graphic will be presented each time during the user’s login.

Examples of password reset questions that could be used in this scheme are shown in Table 1. It would also be a good idea to enable the user to create his/her own question, if they decide that it gives them more security. The final recommendation for a password is as follows: it has to contain at least one digit as well as small and capital letters.

**Figure 3:**
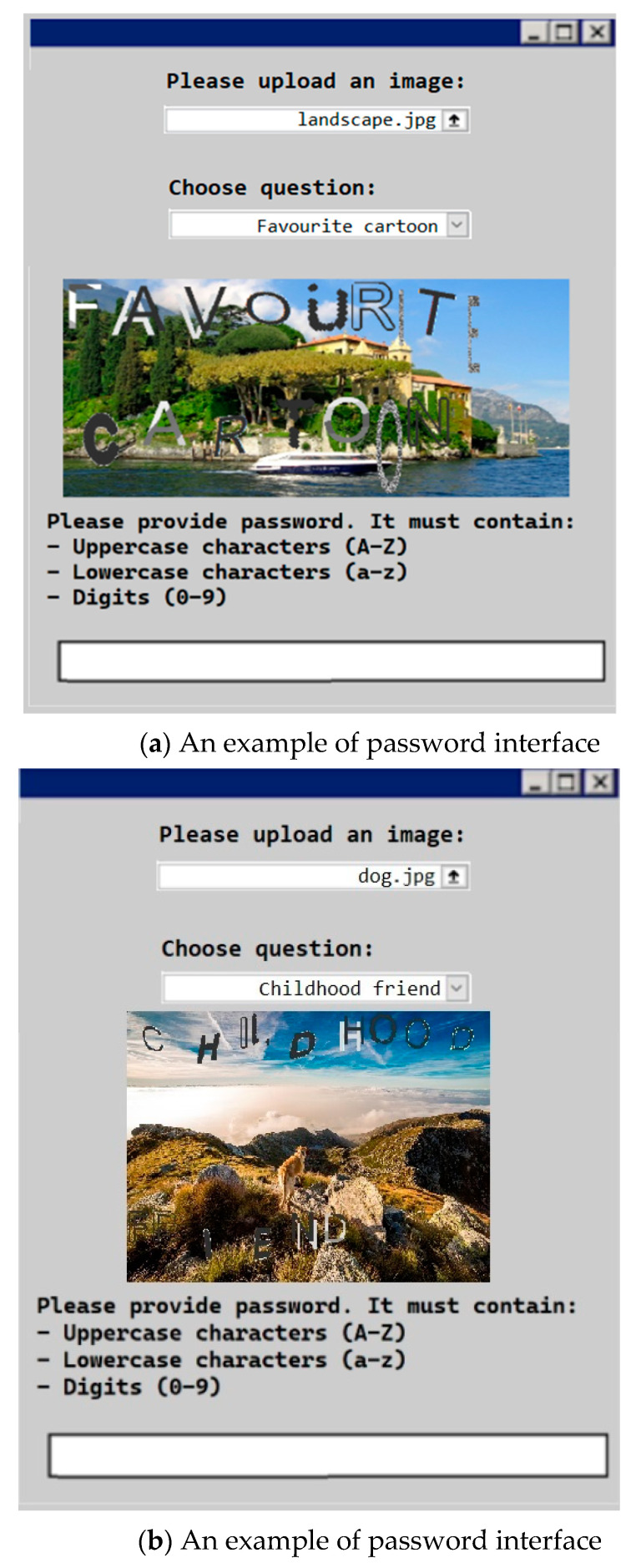
Examples of password creation interface used in the CAPTCHA password reminder scheme.

Figure 3 shows an example of a password creation interface from an application algorithm that generates the CAPTCHA password reminder scheme written in MATLAB 2012a.

## 5. Advantages and Disadvantages of the CAPTCHA Password Reminder Protocol

As has been proven by researchers [13], an emotional, negative, or positive experience with an image activates distinct brain regions responsible for augmenting mnemonic processing, while the emotional content of stimuli can enhance a person’s memory when presented again with the stimuli [13]. Following this, we can expect that when there is a strong emotional connection with an image, be it positive or negative, the chances of the user remembering hidden information from an image are much higher compared to an emotionally irrelevant photo. The main goal of the method described above is to find a memory–visual link which already exists and which is very strong. This is what would differentiate a password created using a CAPTCHA password reminder scheme from a regular password. The image as well as the password are tailored to an individual memory. For the same image and same password reset question, we would probably get as many different passwords as there are users. People’s knowledge, life experience, personal preferences, and most importantly, their visual memory are so unique that it makes sense to exploit them for safety purposes. The CAPTCHA password reminder scheme is one of many possible examples of password reminders. Its main advantage is that it is easy to implement and does not require a password manager or special effort to remember the password. It would only require an additional 1–2 min spent on creating one’s own personal login scheme. 

Uploading an image and picking a question takes a few seconds, associating passwords with them could take a little more time, but in exchange it will stop users from re-using their old passwords or from creating too trivial ones. Should the CAPTCHA password reminder scheme be uploaded by a user on a number of different platforms, it would be enough for that person to pick a different ‘password reset’ question to have several completely different passwords. The ‘image’ part of a password would be often recalled due to multiple use, but the uniqueness of the answer to the question would still guarantee safety between platforms. 

As a downside of the presented scheme, we can count the fact that the service providers would have to slightly increase the memory in the database assigned to a user in order to store their password login scheme. Loading up an image, after a user’s login is provided, probably would not impact the authentication process noticeably (since the image size can be reduced by service providers), but it would require an additional step, since login and passwords cannot be provided at once.

One of the main advantages of the CAPTCHA password reminder scheme is its ability to enable the user to create longer passwords by giving them two reminders: an image and reset question. A typical word in English is around five letters, when multiplying this by two and adding one digit, we can assume that the average password created using the described method would not be shorter than eleven characters. A standard password recommendation is eight to nine characters or longer.

There is a high chance that users may experience a learning curve and will not immediately create a password that is completely non-image related. This, however, can be fixed by applying reverse image search by service providers on uploaded files, and doing a dictionary check on the pages on which the said image was found. In the case when the user’s password matches with a word from the image search, the app should report the problem and request another one.

## 6. Conclusions and Future Work

The purpose of this paper was to present a new CAPTCHA password reminder method, designed to securely manage password storage and to allow users to retrieve passwords more easily by using personalised image patterns and reminder questions. The protocol presented is based on a given user’s experience and knowledge to create or recall a password based on their own memories, experiences, or cognitive associations. 

Our own memories and experiences are the only thing that can stay forever secret and are not accessible by any technology. The harm caused by hackers or by losing passwords is becoming a serious problem, and more advances in all related fields are needed. The method presented in this article focused on implementation of a personal password reminder. The need to have a reminder is clear, as we are close to having around 200 different login credentials. The sooner users learn to create longer and non-trivial passwords, the better their chances of not becoming a victim of a hacker’s attack. Moreover, phishing attempts by providing fake login interface would be stopped, since hackers could not know what a user’s password reminder image looks like.

The described scheme encourages users to search for a special image–word link in their memory, for which they have a mnemonic reaction [14]. The already existing logical path ensures that remembering passwords will be easier in comparison to standard login procedures without any password reminder. Apart from that, the CAPTCHA password reminder scheme tries to force the creation of longer passwords. It is common knowledge that users try to pass account creation and login processes as fast as possible, but we believe that the presented scheme and creation of the password reminder image will take less time with each attempt. Implementation of the presented method is easy and does not require huge computing power or a bigger storage engine [15]. In the presented CAPTCHA password reminder scheme, it is also possible to use several different images for a particular user’s authentication to create this model more efficiently. The application of a multi-image approach can increase the security of the proposed scheme. 

A protocol based on a sequence of images, the order or semantic content which determines the user’s associations, may be a particularly interesting solution. In such protocols, it is possible to reproduce several different passwords separately depending on the sequence of images presented. Changing the sequence will allow only one password to be revealed, and subsequent passwords will be revealed after the user is presented with familiar images, but in a different order.

On the other hand, the application of the same image for the authentication of different persons or groups of users can decrease the security level considerably, because it facilitates password recovery within a user group [16,17,18].

Future work will focus on measuring user-friendliness and on improving the CAPTCHA password reminder scheme. The search for a more robust ‘personal reminder’ is still ongoing, but there are many limitations to this type of technique. Advances in technology and machine learning algorithms can easily decode almost every image, so the strength of every password reminder scheme needs to lie in a very specific knowledge or memory of the account owner.

## Figures and Tables

**Table 1:** Example of standard password reset questions.

Examples of Reset Questions	
Q1	What was the name of your first manager at your first job?
Q2	What high school did you attend?
Q3	What is your mother’s maiden name?
Q4	What is the name of the road you grew up on?

## Data Availability

No new data were created or analysed in this study. Data sharing is not applicable to this article.
